# Short-Term Irisin Treatment Enhanced Neurotrophin Expression Differently in the Hippocampus and the Prefrontal Cortex of Young Mice

**DOI:** 10.3390/ijms24119111

**Published:** 2023-05-23

**Authors:** Manuela Dicarlo, Patrizia Pignataro, Roberta Zerlotin, Clelia Suriano, Chiara Zecca, Maria Teresa Dell’Abate, Giuseppina Storlino, Angela Oranger, Lorenzo Sanesi, Giorgio Mori, Maria Grano, Graziana Colaianni, Silvia Colucci

**Affiliations:** 1 Department of Precision and Regenerative Medicine and Ionian Area, University of Bari, Piazza Giulio Cesare 11, 70124 Bari, Italy; patrizia.pignataro@uniba.it (P.P.); roberta.zerlotin@uniba.it (R.Z.); clelia.suriano@uniba.it (C.S.); angela.oranger@uniba.it (A.O.); maria.grano@uniba.it (M.G.); graziana.colaianni@uniba.it (G.C.); 2 Department of Translational Biomedicine and Neuroscience, University of Bari, 70124 Bari, Italy; lorenzo.sanesi@uniba.it; 3 Center for Neurodegenerative Diseases and the Aging Brain, Department of Clinical Research in Neurology, University of Bari at “Pia Fondazione Card G. Panico” Hospital, Via San Pio X, 4, 73039 Tricase, Italy; chiarazecca.cz@gmail.com (C.Z.); dellabatemariateresa@gmail.com (M.T.D.); 4 Department of Clinical and Experimental Medicine, University of Foggia, 71100 Foggia, Italy; giuseppina.storlino@unifg.it (G.S.); giorgio.mori@unifg.it (G.M.)

**Keywords:** depression, antidepressant, irisin, fibronectin type III domain containing 5, physical exercise, BDNF, NGF, FGF-2, IGF, IL-6, IL-1β, neurotrophins, neuroinflammation, mice

## Abstract

As a result of physical exercise, muscle releases multiple exerkines, such as “irisin”, which is thought to induce pro-cognitive and antidepressant effects. We recently demonstrated in young healthy mice the mitigation of depressive behaviors induced by consecutive 5 day irisin administration. To understand which molecular mechanisms might be involved in such effect, we here studied, in a group of mice previously submitted to a behavioral test of depression, the gene expression of neurotrophins and cytokines in the hippocampus and prefrontal cortex (PFC), two brain areas frequently investigated in the depression pathogenesis. We found significantly increased mRNA levels of nerve growth factor (NGF) and fibroblast growth factor 2 (FGF-2) in the hippocampus and brain-derived growth factor (BDNF) in the PFC. We did not detect a difference in the mRNA levels of interleukin 6 (IL-6) and IL-1β in both brain regions. Except for BDNF in the PFC, two-way ANOVA analysis did not reveal sex differences in the expression of the tested genes. Overall, our data evidenced a site-specific cerebral modulation of neurotrophins induced by irisin treatment in the hippocampus and the PFC, contributing to the search for new antidepressant treatments targeted at single depressive events with short-term protocols.

## 1. Introduction

Physical exercise is widely recognized as a powerful nonpharmacological therapeutic tool by displaying marked health benefits in patients with pathologies involving the cardiovascular, locomotor, respiratory, nervous, and immune systems [1]. Furthermore, irrefutable evidence has proven the effectiveness of regular physical activity in reducing the risk factors associated with various chronic diseases [2]. Several biomolecules released by both muscle and non-muscle tissues in response to physical exercise have been implicated in the molecular pathways underlying the beneficial effects of exercise [3]. Recently, the term “exerkines” has been coined to globally refer to those humoral factors that are directly secreted into the bloodstream or transported by extracellular vehicles (EVs) such as exosomes [4]. Among them, the myokine irisin has gained attention for its role in the crosstalk among muscle and other target tissues including adipose, bone, and nervous tissue [5,6]. Irisin is a 112-amino-acid polypeptide derived from the proteolytic cleavage of the transmembrane glycoprotein fibronectin type III domain-containing protein 5 (FNDC5) following activation of the transcriptional cofactor peroxisome proliferator-activated receptor-gamma coactivator-1-alpha (PGC-1α) in the exercised skeletal muscles [7,8]. It has been reported that physical exercise also increased *FNDC5* expression in the hippocampus through a mechanism that, similarly to skeletal muscles, involved PGC-1α [9]. Interestingly, a number of studies evidenced the role of irisin in the development of the central nervous system (CNS) by participating in neurogenesis and promoting neuronal differentiation [10,11]. In addition, a neuroprotective effect of irisin has been observed for several acute and chronic CNS pathologies (i.e., stroke, neurodegenerative diseases, and psychiatric disorders) [6,11].

As far as psychiatric disorders are concerned, some research groups focused on the beneficial effects of irisin on major depressive disorder (MDD), the most common and severe psychiatric illness impacting an estimated 300 million people around the world [12,13,14,15]. MDD is considered to be a multifactorial disorder; indeed, various causes such as genetic susceptibility, alterations in the neurobiological systems involved in the stress responses (hypothalamic–pituitary–adrenal axis, autonomic nervous system, and immunity system), and environmental stressful events (i.e., employment loss, violence exposure, separation, etc.) have been associated with an increased risk of MDD development [16,17,18,19]. Considering the complex pathogenesis of depressive disorders, several treatment measures have been proposed for patients with depression, including pharmacological, psychological, and neurostimulator therapies [20]. However, a certain proportion of depressed patients did not achieve and/or sustain symptom remission even after serial therapy trials. This condition, known as “treatment-resistant depression” (TRD), is frequently due to the presence of psychiatric comorbidities, especially anxiety, that compromises the treatment response [20,21] These issues led to pharmacological studies devoted to investigating the effects of new antidepressant drugs [22]. Furthermore, to better understand the biological mechanisms involved in human refractory treatment depression, different rodent models of TRD have been proposed [23].

At present, the role of irisin as a potential factor that could exert an antidepressant effect has been described in preclinical studies performed on rodents [14,15]. Interestingly, Wang and Pan first showed that irisin levels were decreased in the brain of rats exposed to chronic unpredictable stress (CUS), a rodent model of depression. Moreover, they demonstrated that irisin subcutaneous administration could reverse the CUS-induced behavioral deficits acting on the regulation of the energy metabolism in the prefrontal cortex (PFC) of the brain [14]. Similarly, Siteneski et al. showed that irisin directly injected into cerebral lateral ventricles decreased the depressive-like behaviors in young healthy male mice by modulating the cerebral gene expression of *Pgc-1α, Fndc5,* and *Bdnf* [15].

In accordance with the abovementioned studies, our group recently demonstrated that systemic administration of irisin via intermittent intraperitoneal injection (100 μg/kg/week for 1 month) was able to reduce depressive-like behaviors in young healthy mice. Specifically, we noted a significant immobility time decrease in two behavioral paradigms commonly used to test potential antidepressant drugs, i.e., the tail suspension test (TST) and the forced swim test (FST). Moreover, we observed that these findings were probably due to changes in the expression of neurotrophic/growth factors such as brain-derived neurotrophic factor (*Bdnf*) and insulin-like growth factor 1 (*Igf*-1) and some cytokines interleukin-1β [(*Il-1β*), interleukin-4 (*Il-4*), interleukin-6 (*Il-6*), and interleukin-10 (*Il-10*)] [24].

The Food and Drug Administration (FDA) suggests the short-term use of new nonselective serotonin reuptake inhibitor (non-SSRI) antidepressants to treat single episodes of depression that often occur in children and adolescent [25]. Thus, in our previous work, we proposed a short-term irisin treatment schedule. Maintaining the same total cumulative dose used in [24], we administered irisin subcutaneously for 5 days consecutively (100 μg/kg/daily) in young healthy female and male mice, and we investigated the effects of irisin on the depressive- and anxiety-like behaviors, as well as memory performance. Of note, we showed that irisin exerted both a significant antidepressant and an anxiolytic effect without obvious sex differences [26].

Here, we aim to shed light on the molecular pathways underlying the intriguing findings regarding irisin’s antidepressant effect by examining the expression of selected genes in cerebral areas considered to be the most implicated in MDD, i.e., the hippocampus and the PFC [27,28,29]. Therefore, in a group of mice already submitted to a behavioral test of depression [26], we examined the expression of some neurotrophic/growth factors associated with the pathogenesis of depressive disorders, such as BDNF, IGF-1, nerve growth factor (NGF), and fibroblast growth factor 2 (FGF-2) [30,31,32,33]. Furthermore, to complete our investigations, we considered irisin’s effect on chronic neuroinflammation, a well-known risk factor for depression [19,34]. For this purpose, we evaluated the expression of a selection of pro- and anti-inflammatory cytokines, i.e., IL-6, IL-1β, IL-10, IL-4, and interleukin-1 receptor antagonist (IL-1ra). 

## 2. Results

### 2.1. Short-Term Irisin Injections Differently Increased the Expression of Neurotrophic/Growth Factors in the Hippocampus and the Prefrontal Cortex (PFC)

**Figure 1:**
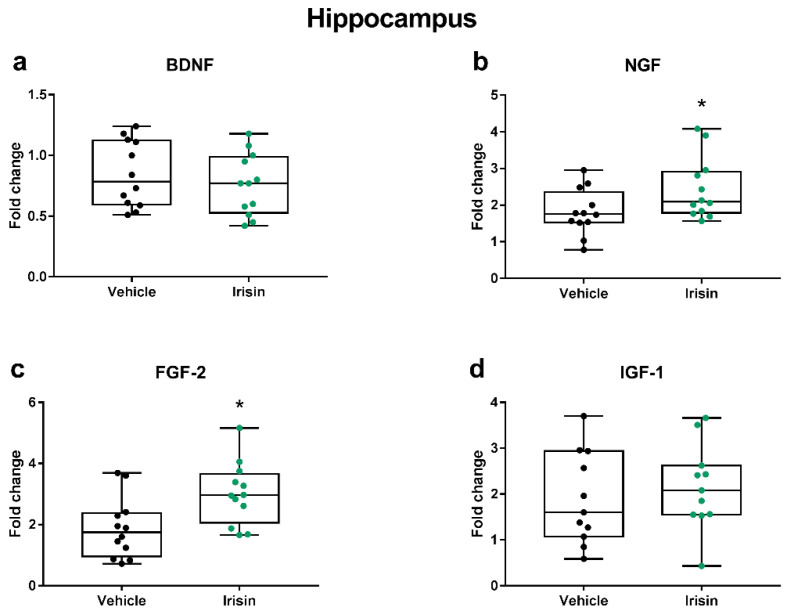
The impact of short-term systemic irisin administration on the expression of some neurotrophic/growth factors in the hippocampus. The gene expression of *Bdnf* (**a**), *Ngf* (**b**), *Fgf-2* (**c**), and *Igf-1* (**d**) was assessed by qRT-PCR in female (*n* = 7) and male (*n* = 5) mice for each treatment group. The Shapiro–Wilk test was followed by Student’s t test for all data except for *Ngf*; * *p* < 0.05. Data are presented as box-and-whisker plots with median and interquartile ranges, from max to min, with all data points shown.

In our recent work, we found that once-daily irisin injections (100 μg/kg) for 5 consecutive days decreased the depressive- and anxiety-like behaviors in young healthy female and male mice [26]. In line with these findings, in this study, with the aim of assessing the impact of short-term irisin administration on the expression of the most well-known neutrophic/growth factors associated with depression [30,31,32,33], we evaluated the gene expression of *Bdnf*, *Igf-1*, *Ngf*, and *Fgf-2* in hippocampal and PFC tissues. We noticed that irisin injections significantly increased *Ngf* and *Fgf-2* expression in the hippocampus of irisin-treated mice (*p* < 0.05), while no differences were observed for *Bdnf* and *Igf-1* (*p* = 0.433 and *p* = 0.554, respectively) (Figure 1).

**Figure 2:**
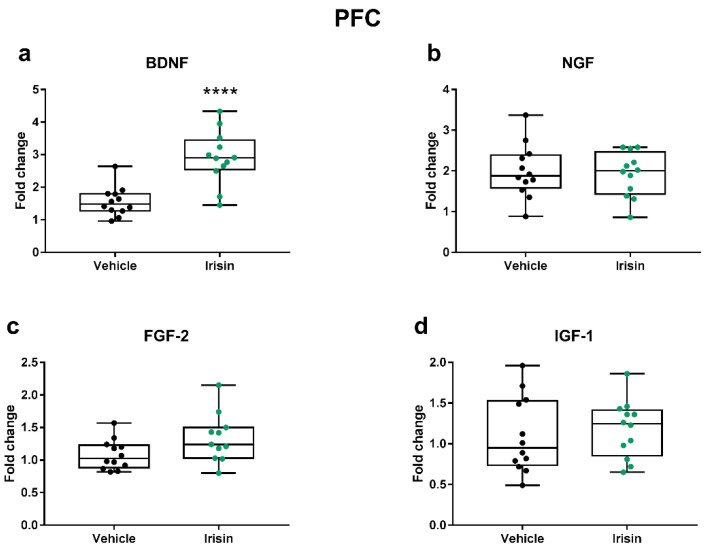
The effect of short-term systemic irisin administration on the expression of some neurotrophic/growth factors in the PFC. The gene expression of *Bdnf* (**a**), *Ngf* (**b**), *Fgf-2* (**c**), and *Igf-1* (**d**) was assessed by qRT-PCR in female (*n* = 7) and male (*n* = 5) mice for each treatment group. The Shapiro–Wilk test was followed by Student’s t-test for all data; **** *p* < 0.0001. Data are presented as box-and-whisker plots with median and interquartile ranges, from max to min, with all data points shown.

In PFC tissue, the short-term irisin injections modulated the expression of the abovementioned genes in a different manner compared to the hippocampus. Indeed, we noticed a considerable increase in BDNF mRNA levels (*p* < 0.0001) and a value near to significance for *Fgf-2* (*p* = 0.059) in irisin-treated mice compared to controls. We found no differences for either *Ngf* or *Igf-1* (*p* = 0.765 and *p* = 0.643, respectively) (Figure 2).

### 2.2. Short-Term Irisin Administration Did Not Affect the Expression of Il-6 and Il-1β in Either the Hippocampus or the PFC

**Figure 3:**
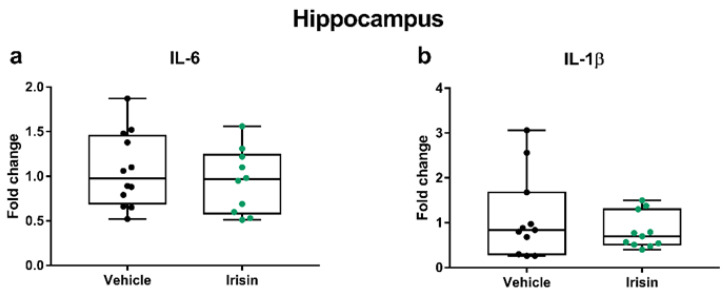
The effect of short-term systemic irisin treatment on cytokine profile in the hippocampus. The gene expression of *Il-6* (**a**), and *Il-1β* (**b**) was evaluated by qRT-PCR in female (*n* = 7) and male (*n* = 5) mice for each treatment group. The Shapiro–Wilk test was followed by Student’s t test for *Il-6* data analysis and by the Mann–Whitney test for *Il-1β*. Data are presented as box-and-whisker plots with median and interquartile ranges, from max to min, with all data points shown.

Extensive clinical studies have shown the role of the immune system in depression; in fact, immune dysfunctions that alter the balance between proinflammatory and anti-inflammatory cytokines have been related to depressive disorders [35]. In accordance with this “cytokine hypothesis of depression”, we examined how short-term irisin injections may affect the cytokine profile in the hippocampus and the PFC by analyzing the expression of pro- (IL-6 and IL-1β) and anti-inflammatory (IL-4, IL-10, and IL-1ra) cytokines majorly involved in MDD. In the hippocampus, we observed no differences for the IL-6 and IL-1β mRNA levels between the irisin-treated mice and controls (*p* = 0.476 and *p* = 0.549, respectively) (Figure 3). 

**Figure 4:**
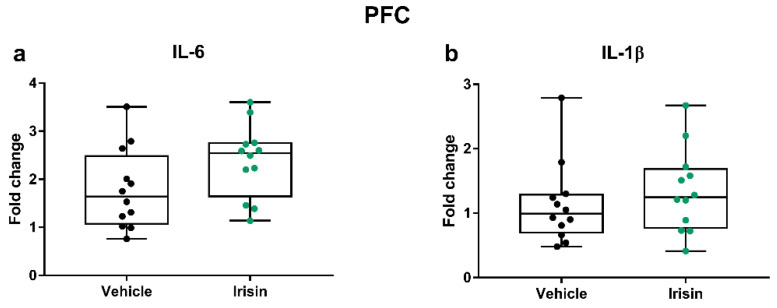
The effect of short-term systemic irisin treatment on cytokine profile in the PFC. The gene expression of *Il-6* (**a**) and *Il-1β* (**b**) was evaluated by qRT-PCR in female (*n* = 7) and male (*n* = 5) mice for each treatment group. The Shapiro–Wilk test was followed by Student’s t test for *Il-6* data analysis and by the Mann–Whitney test for *Il-1β*. Data are presented as box-and-whisker plots with median and interquartile ranges, from max to min, with all data points shown.

In the PFC, we found a slight increase in *Il-6* expression in irisin-treated mice that nonetheless did not reach statistical significance (*p* = 0.081), while no differences were noticed for *Il-1β* (*p* = 0.409) (Figure 4). 

As far as the expression of anti-inflammatory cytokines is concerned, we observed that their mRNA levels were too low for accurate analysis to allow comparisons between the experimental groups. It can be hypothesized that anti-inflammatory cytokines are expressed only under conditions of memory impairment. Therefore, their expression was undetectable in young and healthy mice, and the behavioral paradigm of depression (forced swim test) was not effective in increasing their expression levels.

### 2.3. Short-Term Irisin Administration Modulated Gene Expression in a Sex-Independent Way in Both the Hippocampus and the PFC

**Figure 5:**
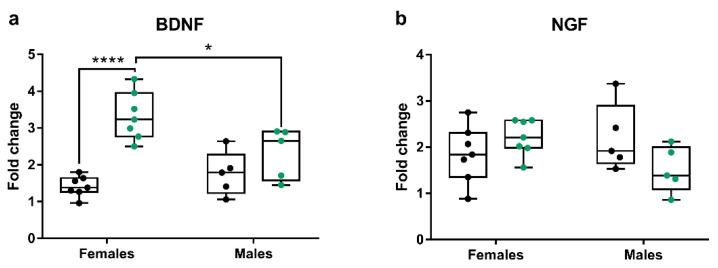
Gene expression of *Bdnf* (**a**) and *Ngf* (**b**) in the PFC of male and female mice injected with irisin for short-term. The Shapiro–Wilk test was followed by two-way ANOVA analysis and Tukey’s multiple comparison test; * *p* < 0.05; **** *p* < 0.0001. Data are presented as box-and-whisker plots with median and interquartile ranges, from max to min, with all data points shown. Black dots indicated vehicle-treated mice; green dots indicated irisin-treated mice.

To investigate the effect of short-term irisin treatment, sex, and their interactions on gene expression in the hippocampus and the PFC, we performed separate group comparisons according to treatment and sex by two-way ANOVA analysis. All the results of this analysis are included in Table 1, Table 2 and Table 3. For the hippocampus, we did not find any significant interaction of treatment and sex for all the analyzed genes. As far as the PFC tissue is concerned, two-way ANOVA analysis showed an interaction of sex and treatment for *Bdnf* and *Ngf* expression (*p* = 0.009 and *p* = 0.034, respectively). A post hoc test revealed that female irisin-treated mice had higher BDNF mRNA levels compared to their control group (F = 8.99, DF:20, *p* < 0.0001) and male irisin-treated mice (F = 4.314, DF:20, *p* < 0.05). No differences were noticed among groups for *Ngf* expression (Figure 5).

## 3. Discussion

In the last decade, the myokine irisin emerged as an interesting neuroprotective factor in the muscle–brain crosstalk thanks to its ability to cross the blood brain barrier and to induce the cerebral expression of neurotrophins [9,36,37].

In our first study, we showed that, in healthy young male mice, treated weekly with irisin for 1 month, depressive-like behaviors were reduced [24]. In line with these results, we recently reported that short-term irisin treatment daily for 5 days also displayed antidepressant effects, as well as a reduction in anxiety-like behaviors, in young healthy female and male mice [26]. Herein, we investigated the molecular basis of the antidepressant effect of short-term irisin treatment analyzing the cerebral expression of the most well-known factors associated with MDD pathogenesis. This study was performed in a group of mice previously submitted to a behavioral paradigm of depression (i.e., forced swim test) displaying high mobility when treated with irisin compared to untreated control mice [26]. Cross-sectional studies using structural neuroimaging in patients with MDD and investigations in rodent models of depression demonstrated the presence of morphological (i.e., volume reduction, reduced synapse number, etc.) and molecular alterations in both the hippocampus and the PFC [19,27,28,38]; thus, we focused our attention on these specific cerebral regions.

The present study revealed that the recent antidepressant effect of irisin could be mediated by a site-specific action on the modulation of neurotrophins and growth factors at the level of the hippocampus and prefrontal cortex (PFC). Indeed, we found that irisin injection once daily for five consecutive days increased the expression of *Ngf* in the hippocampus and that of *Bdnf* in the PFC of irisin-treated mice. NGF and BDNF are two members of the neurotrophic factor family with crucial roles in the formation and plasticity of cerebral neural networks, and they are mainly involved in the pathophysiological mechanism of depression [30,32,39]. In fact, it was reported that depressed patients displayed reduced circulating NGF levels [40]. Regarding BDNF, several evidence found an abnormal decrease in its serum levels in MDD patients, thereby representing a biomarker of the pathogenesis of depression [41,42,43,44]. In addition, it was reported that chronic stress and depressive conditions reduced the expression of *Bdnf* in several brain regions, including the hippocampus and the PFC, in rodent models [30,45,46]. Interestingly, the site-specific modulation of the two neurotrophins induced by short-term irisin administration demonstrated here in the two brain regions is in agreement with studies of Lima-Filho and coworkers, who described a region-specific gene expression induced by antidepressant drugs in mice [47].

Conflicting literature data about the effect of irisin on Bdnf levels in the hippocampus have been reported. The unaltered *Bdnf* modulation in the hippocampus we found is in contrast with the upregulation demonstrated by other authors [9,36], but in line with the observations of Siteneski and colleagues [15], who found an irisin time-dependent modulation of *Bdnf* and other genes. In particular, the latter authors showed that *Bdnf* was downregulated in the hippocampus 1 h after irisin administration and upregulated after 6 h. Therefore, it might be speculated that the unaltered expression of *Bdnf* in the hippocampus we detected might have been due to the short time elapsed since the last irisin injection.

We also studied FGF-2, another neurotrophic factor able to regulate neurogenesis, synaptic formation, and growth [33]. FGF-2 has been implicated in stress-related mood disorders, such as depression, and its expression is reduced in the hippocampus of MDD patients [48,49,50,51]. In this work, we observed a significant increase in the mRNA levels of FGF-2 in irisin-treated mice in the hippocampus and the PFC, suggesting that, unlike the above-discussed neurotrophins, *Fgf-2* did not display a cerebral site-specific increase. Of note, other studies reported the upregulation of *Fgf-2* expression after antidepressant treatments [52,53]. For example, Maragnoli et al. observed higher levels of FGF-2 mRNA in the PFC of rats submitted to short- and long-term treatment with a combination of antidepressant drugs [52].

IGF-1 is a neutrotrophic factor that is expressed by different cells in the CNS [31]. In the synaptic plasticity process, IGF-1 is involved in the control of synapse formation, neurotransmitter release, and neuron excitation [54]. Considering these roles, as well as IGF-1’s ability to suppress neuroinflammation [31], it has been hypothesized that the impairment of IGF-1 functions could be responsible for some clinical alterations observed in MDD patients, including cognitive compromission [31]. In line with this assumption, a reduced *Igf-1* expression was described in the hippocampus and the PFC of prenatally stressed rats that exhibited depressive-like behaviors [55]. Unlike our previous study, in which we found *Igf-1* upregulation in irisin-treated mice receiving a long-term intermittent administration [24], we observed here that short-term irisin treatment seemed to not involve the IGF-1 system as its expression did not change in either the hippocampus or the PFC.

Recently, a number of meta-analysis results and animal studies suggested that the immune system dysregulation and the activation of the inflammatory response system are closely related to depressive disorders [30,56,57,58]. In particular, it was shown that the levels of some inflammatory cytokines (i.e., IL-6, IL-1, TNF-α, etc.) were significantly increased in the serum of MDD patients [56,57] and in the brain of rodent models of depression [58]. Proinflammatory cytokine secretion can contribute to depression by impacting neurotransmitter levels, neurogenesis, neuroendocrine function, neuroplasticity, and depression-related neural networks [30,56,58]. As a result, some anti-inflammatory drugs have been shown to have antidepressant effects or increase the action of antidepressant drugs [30]. In the present study, we analyzed the gene expression of some pro- and anti-inflammatory cytokines and we noticed that there were no differences between the irisin-treated mice and the control group. These observations are in contrast with the results of our previous study in which we observed the effect of irisin in the modulation of different cytokines, such as IL-1β and IL-4 [24]. Collectively, these different findings regarding cytokines, together with the results of *Igf-1* expression, lead us to hypothesize that the modulation of these factors might depend on several conditions such as the time of treatment, i.e., intermittent long-term [24] versus once-daily short-term, and the different brain areas (whole brain versus hippocampus and PFC tissues). However, the complexity of the cytokine system, including a plethora of mediators with multiple effects, requires further in-depth investigations.

Epidemiological findings point to a gender difference in the prevalence and incidence of MDD as depressive disorders affect women more frequently than men [59]. Female hormonal fluctuations and environmental/social factors, such as psychological stress, may be triggers for MDD development [59]. In light of possible different treatment response due to gender, we evaluated the effect of sex on gene expression. Consistently with the behavioral paradigm results [26], no significant differences were noticed between female and male for the analyzed genes except for *Bdnf* expression in the PFC. It is known that the brain expression profile of BDNF is regulated by several factors, including gonadal hormones, estrogens in women, and androgens in men [60]. Sex-related differences in BDNF signaling and function have been observed; indeed, estrogen regulates not only BDNF levels at the gene and protein level, but also those of its receptor in the brain [61,62,63,64], including the hippocampus and PFC [65]. Although some evidence suggests reduced BDNF levels in the PFC in depressed women [66], studies on the subject have been less thorough in the PFC, making the relationship between BDNF and estrogen levels in this part of the brain less clear to date. Similarly, the effects of androgens on BDNF levels have been less studied; hence, further studies on the involvement of sex hormones in the regulation of neurotrophin levels are needed.

Notably, our results showed that short-term irisin treatment markedly increased *Bdnf* expression in female mice compared to males. Collectively, the absence of sex differences, together with the greater response in female mice, is a promising result for desirable clinical applications in humans.

In a recent study by Kim and Leem, the effect of a daily irisin treatment (0.5 μg/kg/day) for seven consecutive days was investigated by evaluating the morphological changes in the hippocampus and the expression of astrocyte-secreted factors associated with neurogenesis [67]. However, to the best of our knowledge, our study is the first to elucidate the molecular mechanisms underlying short-term irisin administration in young mice showing a site-specific expression of neurotrophic factors mainly involved in neurogenesis and/or synaptic plasticity alterations associated with MDD. Noteworthily, our study adds knowledge to the search for new nonselective serotonin reuptake inhibitor (non-SSRI) antidepressant treatments in children and adolescents with MDD, as envisioned by the Food and Drug Administration (FDA) and new regulatory changes encouraging short-term acute efficacy studies [25]. Indeed, we showed here that the expression of neurotrophic factors, such as BDNF, NGF, and FGF-2, which are negatively affected in the pathogenesis of depressive disorders, can be enhanced by short-term treatment with irisin. It is well known that MDD, especially in children and adolescents, is characterized by a disorder that can be sudden and episodic or chronic and recurrent. In fact, great efforts are being made to implement effective pharmacotherapy for the short-term treatment of young people with MDD to understand how to treat a single episode of depression while avoiding the many side-effects of long-term therapies.

## 4. Materials and Methods

### 4.1. Animals

In this study, in order to explore sex-specific treatment responses, male (*n* = 10) and female (*n* = 14) black Swiss mice (C57BL/6) aged 22–25 weeks were used [26]. Animals were housed into six home cages (four per group) 2 weeks before the experiment starting time. Mice had free access to food and water (Harlan Teklad 2019, SDS, England), and they were maintained under 12 h light/dark cycle, controlled noise, and temperature (21 °C). All study procedures were previously approved by the Italian Health Authority (aut. n° 12/2022-PR, obtained on 12 January2022) and conducted in accordance with European Directive no. 2010/63 and with the Italian Regulatory system (D.L. vo n. 26, 4 March 2014). The animal welfare agency (OPBA) of the University of Bari approved all parts of this study concerning animal care and handling protocols.

### 4.2. Short-Term Irisin Administration

Mice were subcutaneously injected with recombinant irisin at 100 µg/kg (*n* = 12) or vehicle (0.9% saline solution sterilized by 0.22 μ filtration) (*n* = 12) once a day for a total of five consecutive days. Recombinant irisin was provided by Adipogen International (San Diego, CA, USA). Treatment was regularly executed at the same time each day. The concentration of irisin was chosen on the basis of previous studies performed by our research team [6,24].

### 4.3. Gene Expression Analysis by qRT-PCR Assays

As described in [26], after the last vehicle/irisin injection, a group of 24 mice consisting of vehicle-treated mice (*n* = 12) and irisin-treated mice (*n* = 12) were submitted to the FST. Each treatment group included *n* = 5 males and *n* = 7 females to assess sex differences. The FST was performed according to the procedure suggested by Porsolt and coworkers [68,69]. Briefly, mice were placed, one at a time, in a clear acrylic cylinder (diameter 13 cm, height 24.5 cm) filled with tap water (18 cm) at 23–25 °C. The immobility time was assessed during 6 min of observation. Mice were considered immobile when they stopped swimming and assumed a passive floating position.

After sacrifice, mouse brains were carefully dissected to isolate the hippocampus and the PFC. These brain tissues were harvested and stored at −80 °C until further analysis. The extraction of total RNA was executed using Trizol (Invitrogen, Carlsbad, CA, USA) according to standard protocols. To synthesize complementary DNA (cDNA), 1 μg of RNA in 20 μL of reaction mixture, i.e., iScript Reverse Transcription Supermix (Bio-Rad Laboratories, Hercules, CA, USA), was used. The expression of mRNAs was measured by qRT-PCR in a 10 μL reaction volume consisting of cDNA transcripts, primer pairs, and SsoFast EvaGreen Supermix (Bio-Rad Laboratories, Hercules, CA, USA). All primers were designed by Primer Blast (https://www.ncbi.nlm.nih.gov/tools/primer-blast/, accessed on 10 January 2023). Glyceraldehyde-3-phosphate dehydrogenase (*Gapdh*) was selected as the reference gene. The sequences for target and reference genes are included in Table 4. To assess the specificity of qRT-PCR products, a melt curve analysis was performed. All samples were assayed in triplicate and quantifications were normalized to *Gapdh* in each reaction.

### 4.4. Statistical Analysis

Data were presented as box-and-whisker plots with median and interquartile ranges, from max to min, with all data points shown, and analyzed using the GraphPad Prism statistical software release 7.0. Data normality was initially verified using the Shapiro–Wilk test. Statistically significant differences between vehicle (control) and irisin-treated mice were evaluated using Student’s *t*-test or the Mann–Whitney test. Statistical significance was set at *p* < 0.05. To determine the effects of irisin treatment (first factor) and sex (second factor) on gene expression, two-way ANOVA was used. Main effects with a *p*-value <0.05 were considered statistically significant. A post hoc Tukey’s multiple comparison test and Student’s *t*-test were used to analyze the differences among the groups.

## 5. Conclusions

Recent studies have evidenced that several defects in the synaptic plasticity (i.e., dendritogenesis, neurogenesis, and axon branching) in some brain regions, including the hippocampus and the PFC, are due to the reduced expression of neurotrophic factors in MDD [39,50,70]. Our data demonstrated that short-term irisin treatment was effective in increasing the cerebral expression of neutrophins in the hippocampus and/or the PFC in young healthy mice previously submitted to a behavioral paradigm of depression. This observation is in line with recent studies evidencing that the neuroprotective effect of antidepressant drugs is mediated, at least in part, by the stimulation of crucial molecular factors involved in brain neurogenesis and/or neural plasticity in both the hippocampus and the PFC [19,71,72]. Taking into account the better behavioral performances of irisin-treated mice in the depression test FST [26], we demonstrated here that exogenous irisin might be responsible for the antidepressant effect in both female and male mice receiving short-term irisin treatment. However, as we observed that the peripheral irisin increase significantly enhanced *Fndc5* and *Pgc-1* expression in both the hippocampus and the PFC [26], we cannot exclude that the activation of the FNDC5/irisin pathway in these regions could contribute to the antidepressant effect of irisin.

## Figures and Tables

**Table 1:** Summary of the statistical analysis for the factors treatment, sex, and treatment × sex by two-way ANOVA.

Brain Regions	Genes	Factors		
		Treatment	Sex	Treatment × Sex
Hippocampus	*Bdnf*	F (1,20) = 1.058; *p* = 0.316	F (1,20) = 4.33; *p* = 0.051	F (1,20) = 1.177; *p* = 0.291
	*Ngf*	F (1,20) = 3.493; *p* = 0.076	F (1,20) = 0.259; *p* = 0.616	F (1,20) = 0.359; *p* = 0.556
	*Fgf-2*	**F (1,20) = 7.395; *p* = 0.013**	F (1,20) = 0.233; *p* = 0.634	F (1,20) = 0.267; *p* = 0.611
	*Igf-1*	F (1,18) = 0.633; *p* = 0.437	F (1,18) = 0.00292; *p* = 0.956	F (1,18) = 1.345; *p* = 0.261
	*Il-6*	F (1,18) = 0.739; *p* = 0.401	F (1,18) = 2.685; *p* = 0.119	F (1,18) = 0.018; *p* = 0.893
	*Il-1β*	F (1,18) = 1.452; *p* = 0.244	F (1,18) = 0.485; *p* = 0.495	F (1,18) = 1.002; *p* = 0.330
PFC	*Bdnf*	**F (1,20) = 28.13; *p* < 0.0001**	F (1,20) = 2; *p* = 0.173	**F (1,20) = 8.413; *p* = 0.009**
	*Ngf*	F (1,20) = 0.492; *p* = 0.491	F (1,20) = 0.538; *p* = 0.472	**F (1,20) = 5.154; *p* = 0.034**
	*Fgf-2*	F (1,19) = 3.464; *p* = 0.078	F (1,19) = 0.596; *p* = 0.449	F (1,19) = 0.029; *p* = 0.866
	*Igf-1*	F (1,20) = 0.300; *p* = 0.589	F (1,20) = 1.364; *p* = 0.257	F (1,20) = 0.282; *p* = 0.601
	*Il-6*	F (1,20) = 3.526; *p* = 0.075	F (1,20) = 0.025; *p* = 0.876	F (1,20) = 0.625; *p* = 0.439
	*Il-1β*	F (1,20) = 0.347; *p* = 0.562	F (1,20) = 1.182; *p* = 0.289	F (1,20) = 1.519; *p* = 0.232

Significant effects are in bold letters.

**Table 2:** Summary of the results of Student’s *t*-tests of gene expression data in the hippocampus.

Gene	Comparison	*t* Value	Df	*p* Value
*Bdnf*	Female vehicle vs. female irisin	*t* = 0.050	12	0.960
	Female vehicle vs. male vehicle	***t* = 2.402**	**10**	**0.037**
	Female vehicle vs. male irisin	*t* = 0.699	10	0.500
	Female irisin vs. male vehicle	***t* = 2.361**	**10**	**0.040**
	Female irisin vs. male irisin	*t* = 0.662	10	0.523
	Male vehicle vs. male irisin	*t* = 1.173	8	0.275
*Ngf*	Female vehicle vs. female irisin	*t* = 2.120	12	0.056
	Female vehicle vs. male vehicle	*t* = 0.957	10	0.361
	Female vehicle vs. male irisin	*t* = 1.766	10	0.108
	Female irisin vs. male vehicle	*t* = 0.919	10	0.379
	Female irisin vs. male irisin	*t* = 0.055	10	0.957
	Male vehicle vs. male irisin	*t* = 0.735	8	0.484
*Fgf-2*	Female vehicle vs. female irisin	*t* = 1.463	12	0.169
	Female vehicle vs. male vehicle	*t* = 0.729	10	0.483
	Female vehicle vs. male irisin	*t* = 1.564	10	0.149
	Female irisin vs. male vehicle	***t* = 2.291**	**10**	**0.045**
	Female irisin vs. male irisin	*t* = 0.023	10	0.982
	Male vehicle vs. male irisin	***t* = 3.122**	**8**	**0.014**
*Igf-1*	Female vehicle vs. female irisin	*t* = 0.266	11	0.795
	Female vehicle vs. male vehicle	*t* = 0.762	9	0.466
	Female vehicle vs. male irisin	*t* = 0.529	8	0.612
	Female irisin vs. male vehicle	*t* = 0.596	10	0.565
	Female irisin vs. male irisin	*t* = 0.885	9	0.399
	Male vehicle vs. male irisin	*t* = 1.449	7	0.191
*Il-6*	Female vehicle vs. female irisin	*t* = 0.711	10	0.493
	Female vehicle vs. male vehicle	*t* = 1.026	10	0.329
	Female vehicle vs. male irisin	*t* = 0.511	10	0.620
	Female irisin vs. male vehicle	*t* = 2.069	8	0.072
	Female irisin vs. male irisin	*t* = 1.355	8	0.213
	Male vehicle vs. male irisin	*t* = 0.513	8	0.622
*Il-1β*	Female vehicle vs. female irisin	*t* = 0.179	11	0.861
	Female vehicle vs. male vehicle	*t* = 0.910	9	0.386
	Female vehicle vs. male irisin	*t* = 0.433	10	0.674
	Female irisin vs. male vehicle	*t* = 1.128	8	0.292
	Female irisin vs. male irisin	*t* = 0.388	9	0.707
	Male vehicle vs. male irisin	*t* = 1.247	7	0.253

Significant effects are in bold letters. Abbreviations: df, degrees of freedom.

**Table 3:** Summary of the results of Student’s *t*-tests of gene expression data in the PFC.

Gene	Comparison	*t* Value	df	*p* Value
*Fgf-2*	Female vehicle vs. female irisin	***t* = 2.427**	**11**	**0.034**
	Female vehicle vs. male vehicle	*t* = 0.932	10	0.373
	Female vehicle vs. male irisin	*t* = 1.734	10	0.114
	Female irisin vs. male vehicle	*t* = 0.855	9	0.415
	Female irisin vs. male irisin	*t* = 0.339	9	0.743
	Male vehicle vs. male irisin	*t* = 0.834	8	0.429
*Igf-1*	Female vehicle vs. female irisin	*t* = 0.012	12	0.991
	Female vehicle vs. male vehicle	*t* = 1.082	10	0.305
	Female vehicle vs. male irisin	*t* = 0.412	10	0.689
	Female irisin vs. male vehicle	*t* = 1.301	10	0.223
	Female irisin vs. male irisin	*t* = 0.514	10	0.619
	Male vehicle vs. male irisin	*t* = 0.808	8	0.442
*Il-6*	Female vehicle vs. female irisin	*t* = 0.863	12	0.405
	Female vehicle vs. male vehicle	*t* = 0.424	10	0.681
	Female vehicle vs. male irisin	*t* = 1.212	10	0.253
	Female irisin vs. male vehicle	*t* = 1.583	10	0.145
	Female irisin vs. male irisin	*t* = 0.712	10	0.493
	Male vehicle vs. male irisin	*t* = 1.688	8	0.130
*Il-1β*	Female vehicle vs. female irisin	*t* = 1.223	12	0.245
	Female vehicle vs. male vehicle	*t* = 0.098	10	0.924
	Female vehicle vs. male irisin	*t* = 0.320	10	0.755
	Female irisin vs. male vehicle	*t* = 1.332	10	0.212
	Female irisin vs. male irisin	*t* = 1.740	10	0.112
	Male vehicle vs. male irisin	*t* = 0.595	8	0.569

Significant effects are in bold letters. Abbreviations: df, degrees of freedom.

**Table 4:** Primer sequences used for quantitative real-time PCR.

Gene Name	Gene Bank Number	Primer Sequence (5′–3′)	Product Size (bp)	Annealing Temperature (°C)
*Gapdh*	NM_001289726.1	Forward ACACCAGTAGACTCCACGACA Reverse ACGGCAAATTCAACGGCACAG	145	60.48 62.59
*Bdnf*	NM_001048139.1	Forward TGAAGTTGGCTTCCTAGCGG Reverse CCTGGTGGAACTTCTTTGCG	146	60.04 59.41
*Ngf*	NM_001112698.2	Forward GGAGCGCATCGAGTTTTGG Reverse CCTCACTGCGGCCAGTATAG	136	59.57 59.97
*Fgf-2*	NM_008006.2	Forward GCTGCTGGCTTCTAAGTGTG Reverse GTCCAGGTCCCGTTTTGGAT	158	59.20 59.96
*Igf-1*	NM_001111276.1	Forward TGCCTGGGTGTCCAAATGTA Reverse TGTATCTTTATTGCAGGTGCGG	170	59.23 59.06
*Il-6*	NM_001314054.1	Forward CCAAGAGATAAGCTGGAGTCACA Reverse CGCACTAGGTTTGCCGAGTA	121	59.80 60.11
*Il-1β*	NM_008361.4	Forward TGCCACCTTTTGACAGTGATG Reverse ATGTGCTGCTGCGAGATTTG	136	59.04 59.55
*Il-4*	NM_021283.2	Forward TCACAGCAACGAAGAACACCA Reverse CAGGCATCGAAAAGCCCGAA	158	60.41 61.31
*Il-10*	NM_010548.2	Forward GTAGAAGTGATGCCCCAGGC Reverse CACCTTGGTCTTGGAGCTTATT	187	60.46 58.31
*Il-1ra*	NM_001039701.3	Forward GTGGCCTCGGGATGGAAAT Reverse TGGTTAGTATCCCAGATTCTGAAGG	148	59.77 59.63

## Data Availability

Not applicable.
